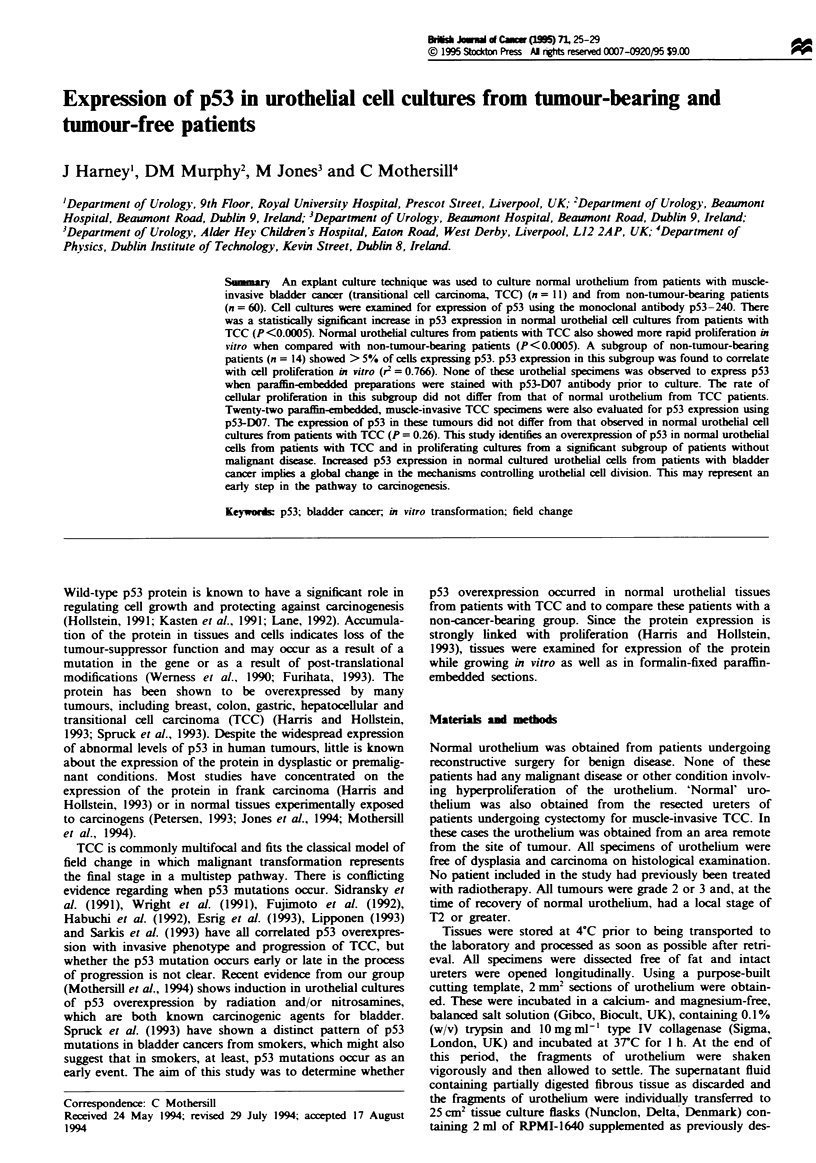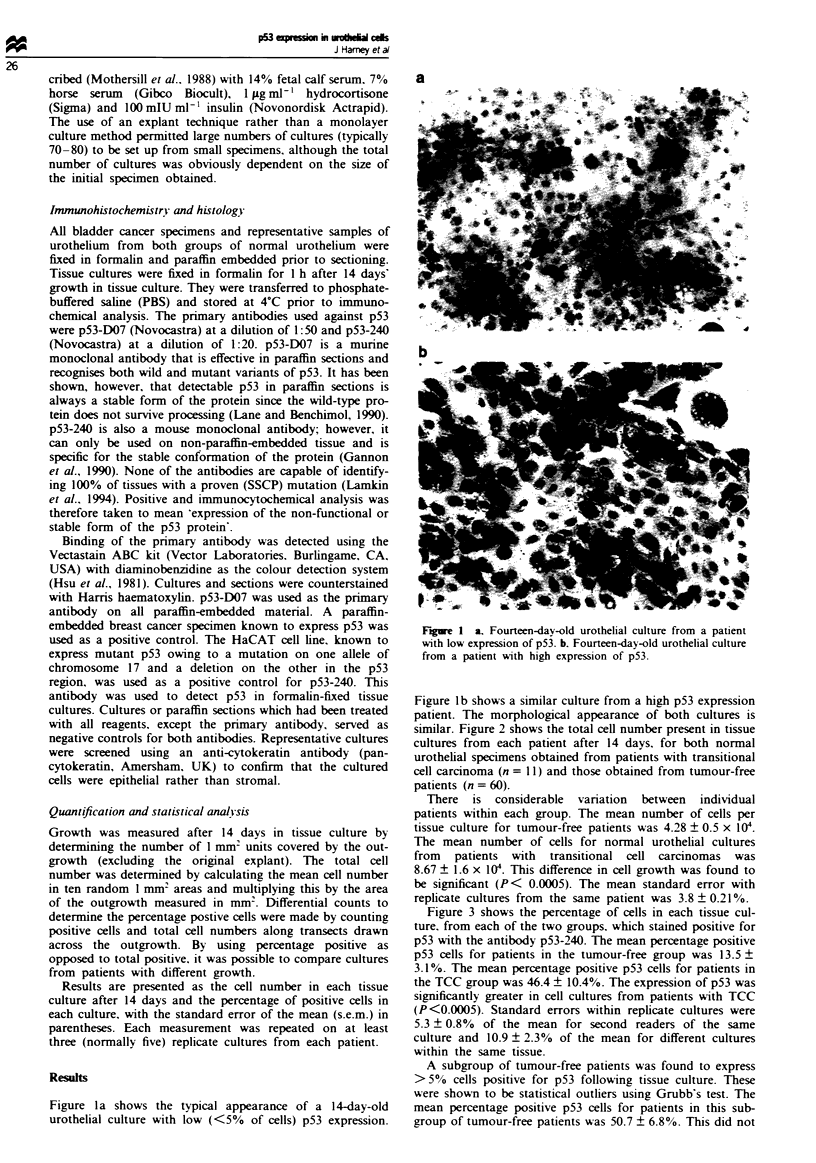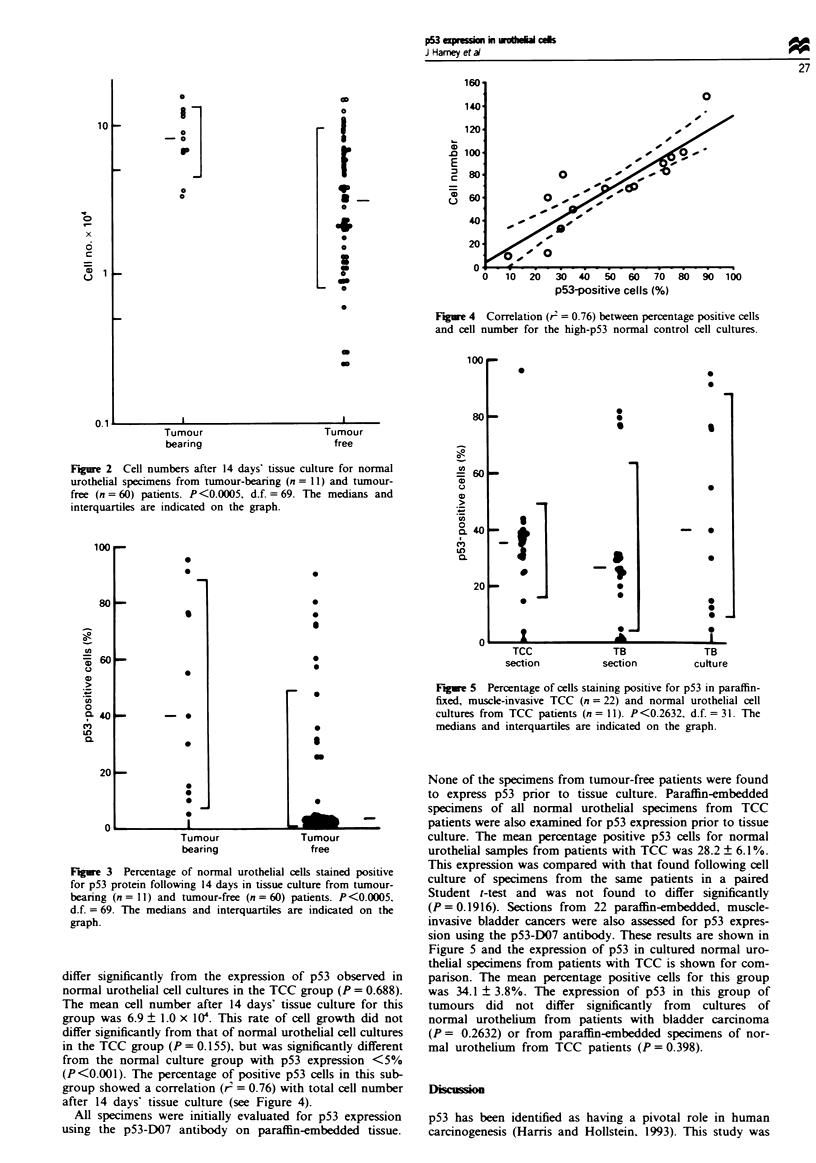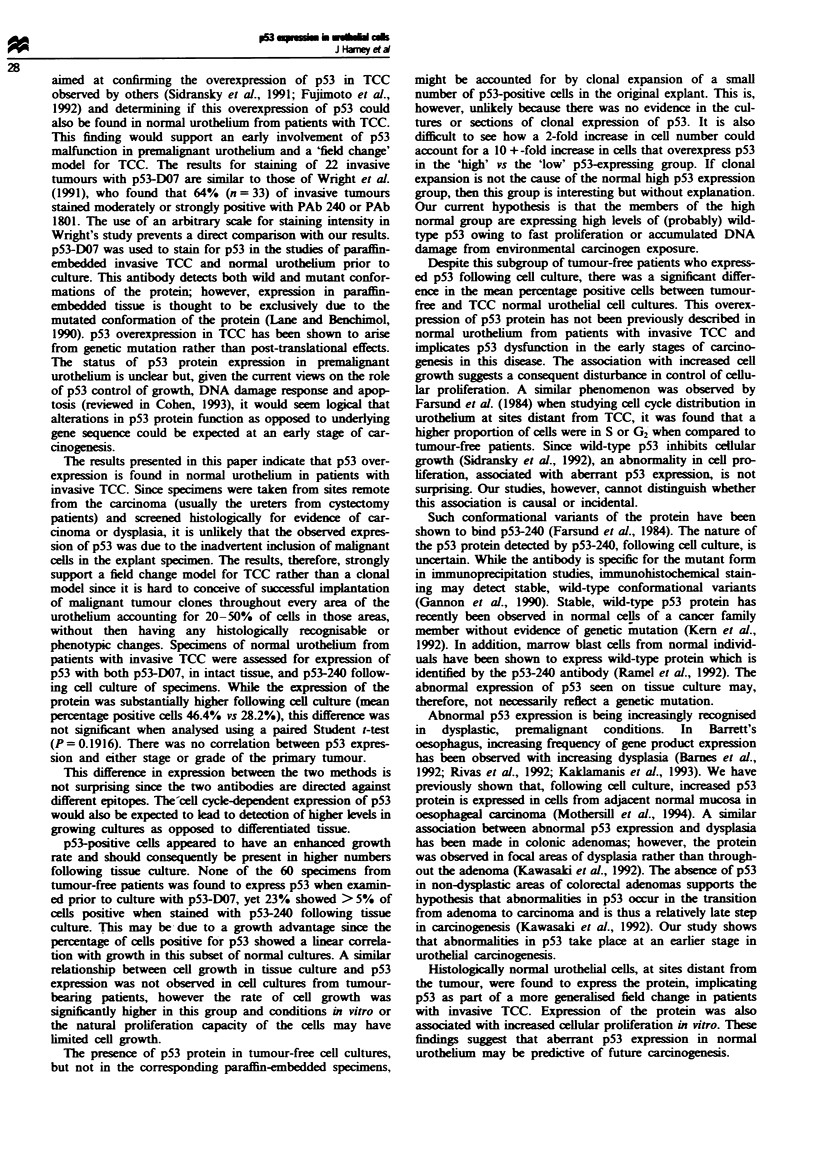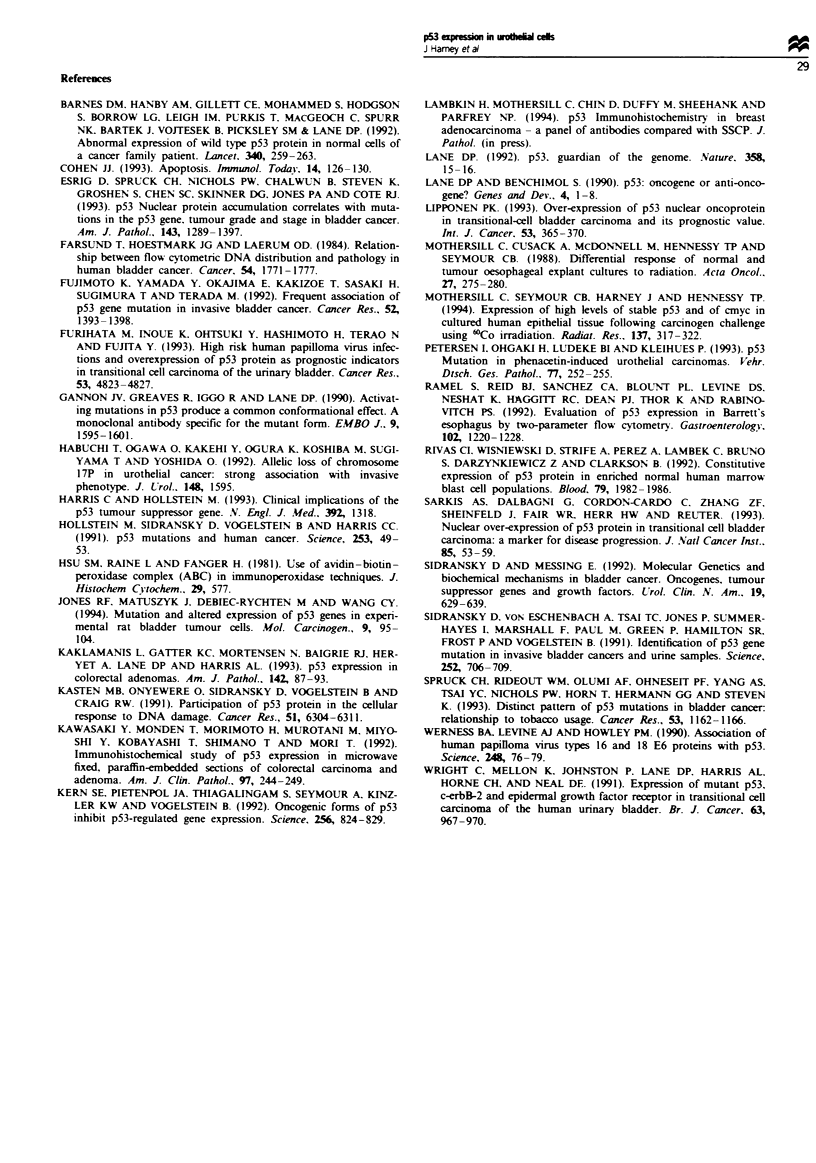# Expression of p53 in urothelial cell cultures from tumour-bearing and tumour-free patients.

**DOI:** 10.1038/bjc.1995.6

**Published:** 1995-01

**Authors:** J. Harney, D. M. Murphy, M. Jones, C. Mothersill

**Affiliations:** Department of Urology, Royal University Hospital, Liverpool, UK.

## Abstract

**Images:**


© 1995 Stockton Press All rights reserved 0007-0920/95 $9.00

### Expression of p53 in urothelial cell cultures from tumour-bearing and tumour-free patients

J Harney[1], DM Murphy[2], M Jones[3] and C Mothersill[4]

[1]*Department of Urology, 9th Floor, Royal University Hospital, Prescot Street, Liverpool, UK;* [2]*Department of Urology, Beaumont Hospital, Beaumont Road, Dublin 9, Ireland;* [3]*Department of Urology, Beaumont Hospital, Beaumont Road, Dublin 9, Ireland;* [3]*Department of Urology, Alder Hey Children's Hospital, Eaton Road, West Derby, Liverpool, L12 2AP, UK;* [4]*Department of Physics, Dublin Institute of Technology, Kevin Street, Dublin 8, Ireland.*

**Summary** An explant culture technique was used to culture normal urothelium from patients with muscle-invasive bladder cancer (transitional cell carcinoma, TCC) ($n = 11$) and from non-tumour-bearing patients ($n = 60$). Cell cultures were examined for expression of p53 using the monoclonal antibody p53-240. There was a statistically significant increase in p53 expression in normal urothelial cell cultures from patients with TCC ($P<0.0005$). Normal urothelial cultures from patients with TCC also showed more rapid proliferation *in vitro* when compared with non-tumour-bearing patients ($P<0.0005$). A subgroup of non-tumour-bearing patients ($n = 14$) showed $>5\%$ of cells expressing p53. p53 expression in this subgroup was found to correlate with cell proliferation *in vitro* ($r^2 = 0.766$). None of these urothelial specimens was observed to express p53 when paraffin-embedded preparations were stained with p53-D07 antibody prior to culture. The rate of cellular proliferation in this subgroup did not differ from that of normal urothelium from TCC patients. Twenty-two paraffin-embedded, muscle-invasive TCC specimens were also evaluated for p53 expression using p53-D07. The expression of p53 in these tumours did not differ from that observed in normal urothelial cell cultures from patients with TCC ($P = 0.26$). This study identifies an overexpression of p53 in normal urothelial cells from patients with TCC and in proliferating cultures from a significant subgroup of patients without malignant disease. Increased p53 expression in normal cultured urothelial cells from patients with bladder cancer implies a global change in the mechanisms controlling urothelial cell division. This may represent an early step in the pathway to carcinogenesis.

**Keywords:** p53; bladder cancer; *in vitro* transformation; field change

Wild-type p53 protein is known to have a significant role in regulating cell growth and protecting against carcinogenesis (Hollstein, 1991; Kasten *et al.*, 1991; Lane, 1992). Accumulation of the protein in tissues and cells indicates loss of the tumour-suppressor function and may occur as a result of a mutation in the gene or as a result of post-translational modifications (Werness *et al.*, 1990; Furihata, 1993). The protein has been shown to be overexpressed by many tumours, including breast, colon, gastric, hepatocellular and transitional cell carcinoma (TCC) (Harris and Hollstein, 1993; Spruck *et al.*, 1993). Despite the widespread expression of abnormal levels of p53 in human tumours, little is known about the expression of the protein in dysplastic or premalignant conditions. Most studies have concentrated on the expression of the protein in frank carcinoma (Harris and Hollstein, 1993) or in normal tissues experimentally exposed to carcinogens (Petersen, 1993; Jones *et al.*, 1994; Mothersill *et al.*, 1994).

TCC is commonly multifocal and fits the classical model of field change in which malignant transformation represents the final stage in a multistep pathway. There is conflicting evidence regarding when p53 mutations occur. Sidransky *et al.* (1991), Wright *et al.* (1991), Fujimoto *et al.* (1992), Habuchi *et al.* (1992), Esrig *et al.* (1993), Lipponen (1993) and Sarkis *et al.* (1993) have all correlated p53 overexpression with invasive phenotype and progression of TCC, but whether the p53 mutation occurs early or late in the process of progression is not clear. Recent evidence from our group (Mothersill *et al.*, 1994) shows induction in urothelial cultures of p53 overexpression by radiation and/or nitrosamines, which are both known carcinogenic agents for bladder. Spruck *et al.* (1993) have shown a distinct pattern of p53 mutations in bladder cancers from smokers, which might also suggest that in smokers, at least, p53 mutations occur as an early event. The aim of this study was to determine whether p53 overexpression occurred in normal urothelial tissues from patients with TCC and to compare these patients with a non-cancer-bearing group. Since the protein expression is strongly linked with proliferation (Harris and Hollstein, 1993), tissues were examined for expression of the protein while growing *in vitro* as well as in formalin-fixed paraffin-embedded sections.

Correspondence: C Mothersill
Received 24 May 1994; revised 29 July 1994; accepted 17 August 1994

### Materials and methods

Normal urothelium was obtained from patients undergoing reconstructive surgery for benign disease. None of these patients had any malignant disease or other condition involving hyperproliferation of the urothelium. 'Normal' urothelium was also obtained from the resected ureters of patients undergoing cystectomy for muscle-invasive TCC. In these cases the urothelium was obtained from an area remote from the site of tumour. All specimens of urothelium were free of dysplasia and carcinoma on histological examination. No patient included in the study had previously been treated with radiotherapy. All tumours were grade 2 or 3 and, at the time of recovery of normal urothelium, had a local stage of T2 or greater.

Tissues were stored at 4°C prior to being transported to the laboratory and processed as soon as possible after retrieval. All specimens were dissected free of fat and intact ureters were opened longitudinally. Using a purpose-built cutting template, 2 $mm^2$ sections of urothelium were obtained. These were incubated in a calcium- and magnesium-free, balanced salt solution (Gibco, Biocult, UK), containing 0.1% (w/v) trypsin and 10 mg $ml^{-1}$ type IV collagenase (Sigma, London, UK) and incubated at 37°C for 1 h. At the end of this period, the fragments of urothelium were shaken vigorously and then allowed to settle. The supernatant fluid containing partially digested fibrous tissue as discarded and the fragments of urothelium were individually transferred to 25 $cm^2$ tissue culture flasks (Nunclon, Delta, Denmark) containing 2 ml of RPMI-1640 supplemented as previously described (Mothersill *et al*., 1988) with 14% fetal calf serum, 7% horse serum (Gibco Biocult), 1 $\mu g\ ml^{-1}$ hydrocortisone (Sigma) and 100 mIU $ml^{-1}$ insulin (Novonordisk Actrapid). The use of an explant technique rather than a monolayer culture method permitted large numbers of cultures (typically 70–80) to be set up from small specimens, although the total number of cultures was obviously dependent on the size of the initial specimen obtained.

### *Immunohistochemistry and histology*

All bladder cancer specimens and representative samples of urothelium from both groups of normal urothelium were fixed in formalin and paraffin embedded prior to sectioning. Tissue cultures were fixed in formalin for 1 h after 14 days' growth in tissue culture. They were transferred to phosphate-buffered saline (PBS) and stored at 4°C prior to immunochemical analysis. The primary antibodies used against p53 were p53-D07 (Novocastra) at a dilution of 1:50 and p53-240 (Novocastra) at a dilution of 1:20. p53-D07 is a murine monoclonal antibody that is effective in paraffin sections and recognises both wild and mutant variants of p53. It has been shown, however, that detectable p53 in paraffin sections is always a stable form of the protein since the wild-type protein does not survive processing (Lane and Benchimol, 1990). p53-240 is also a mouse monoclonal antibody; however, it can only be used on non-paraffin-embedded tissue and is specific for the stable conformation of the protein (Gannon *et al*., 1990). None of the antibodies are capable of identifying 100% of tissues with a proven (SSCP) mutation (Lamkin *et al*., 1994). Positive and immunocytochemical analysis was therefore taken to mean 'expression of the non-functional or stable form of the p53 protein'.

Binding of the primary antibody was detected using the Vectastain ABC kit (Vector Laboratories, Burlingame, CA, USA) with diaminobenzidine as the colour detection system (Hsu *et al*., 1981). Cultures and sections were counterstained with Harris haematoxylin. p53-D07 was used as the primary antibody on all paraffin-embedded material. A paraffin-embedded breast cancer specimen known to express p53 was used as a positive control. The HaCAT cell line, known to express mutant p53 owing to a mutation on one allele of chromosome 17 and a deletion on the other in the p53 region, was used as a positive control for p53-240. This antibody was used to detect p53 in formalin-fixed tissue cultures. Cultures or paraffin sections which had been treated with all reagents, except the primary antibody, served as negative controls for both antibodies. Representative cultures were screened using an anti-cytokeratin antibody (pan-cytokeratin, Amersham, UK) to confirm that the cultured cells were epithelial rather than stromal.

### *Quantification and statistical analysis*

Growth was measured after 14 days in tissue culture by determining the number of 1 $mm^2$ units covered by the outgrowth (excluding the original explant). The total cell number was determined by calculating the mean cell number in ten random 1 $mm^2$ areas and multiplying this by the area of the outgrowth measured in $mm^2$. Differential counts to determine the percentage postive cells were made by counting positive cells and total cell numbers along transects drawn across the outgrowth. By using percentage positive as opposed to total positive, it was possible to compare cultures from patients with different growth.

Results are presented as the cell number in each tissue culture after 14 days and the percentage of positive cells in each culture, with the standard error of the mean (s.e.m.) in parentheses. Each measurement was repeated on at least three (normally five) replicate cultures from each patient.

### Results

Figure 1a shows the typical appearance of a 14-day-old urothelial culture with low (<5% of cells) p53 expression.

a b

**Figure 1** **a**, Fourteen-day-old urothelial culture from a patient with low expression of p53. **b**, Fourteen-day-old urothelial culture from a patient with high expression of p53.

Figure 1b shows a similar culture from a high p53 expression patient. The morphological appearance of both cultures is similar. Figure 2 shows the total cell number present in tissue cultures from each patient after 14 days, for both normal urothelial specimens obtained from patients with transitional cell carcinoma ($n = 11$) and those obtained from tumour-free patients ($n = 60$).

There is considerable variation between individual patients within each group. The mean number of cells per tissue culture for tumour-free patients was $4.28 \pm 0.5 \times 10^4$. The mean number of cells for normal urothelial cultures from patients with transitional cell carcinomas was $8.67 \pm 1.6 \times 10^4$. This difference in cell growth was found to be significant ($P < 0.0005$). The mean standard error with replicate cultures from the same patient was $3.8 \pm 0.21\%$.

Figure 3 shows the percentage of cells in each tissue culture, from each of the two groups, which stained positive for p53 with the antibody p53-240. The mean percentage positive p53 cells for patients in the tumour-free group was $13.5 \pm 3.1\%$. The mean percentage positive p53 cells for patients in the TCC group was $46.4 \pm 10.4\%$. The expression of p53 was significantly greater in cell cultures from patients with TCC ($P < 0.0005$). Standard errors within replicate cultures were $5.3 \pm 0.8\%$ of the mean for second readers of the same culture and $10.9 \pm 2.3\%$ of the mean for different cultures within the same tissue.

A subgroup of tumour-free patients was found to express >5% cells positive for p53 following tissue culture. These were shown to be statistical outliers using Grubb's test. The mean percentage positive p53 cells for patients in this subgroup of tumour-free patients was $50.7 \pm 6.8\%$. This did not

**Figure 2** Cell numbers after 14 days' tissue culture for normal urothelial specimens from tumour-bearing ($n = 11$) and tumour-free ($n = 60$) patients. $P<0.0005$, d.f. = 69. The medians and interquartiles are indicated on the graph.

**Figure 3** Percentage of normal urothelial cells stained positive for p53 protein following 14 days in tissue culture from tumour-bearing ($n = 11$) and tumour-free ($n = 60$) patients. $P<0.0005$, d.f. = 69. The medians and interquartiles are indicated on the graph.

differ significantly from the expression of p53 observed in normal urothelial cell cultures in the TCC group ($P = 0.688$). The mean cell number after 14 days' tissue culture for this group was $6.9 \pm 1.0 \times 10^4$. This rate of cell growth did not differ significantly from that of normal urothelial cell cultures in the TCC group ($P = 0.155$), but was significantly different from the normal culture group with p53 expression <5% ($P<0.001$). The percentage of positive p53 cells in this subgroup showed a correlation ($r^2 = 0.76$) with total cell number after 14 days' tissue culture (see Figure 4).

All specimens were initially evaluated for p53 expression using the p53-D07 antibody on paraffin-embedded tissue.

**Figure 4** Correlation ($r^2 = 0.76$) between percentage positive cells and cell number for the high-p53 normal control cell cultures.

**Figure 5** Percentage of cells staining positive for p53 in paraffin-fixed, muscle-invasive TCC ($n = 22$) and normal urothelial cell cultures from TCC patients ($n = 11$). $P<0.2632$, d.f. = 31. The medians and interquartiles are indicated on the graph.

None of the specimens from tumour-free patients were found to express p53 prior to tissue culture. Paraffin-embedded specimens of all normal urothelial specimens from TCC patients were also examined for p53 expression prior to tissue culture. The mean percentage positive p53 cells for normal urothelial samples from patients with TCC was $28.2 \pm 6.1$%. This expression was compared with that found following cell culture of specimens from the same patients in a paired Student $t$-test and was not found to differ significantly ($P = 0.1916$). Sections from 22 paraffin-embedded, muscle-invasive bladder cancers were also assessed for p53 expression using the p53-D07 antibody. These results are shown in Figure 5 and the expression of p53 in cultured normal urothelial specimens from patients with TCC is shown for comparison. The mean percentage positive cells for this group was $34.1 \pm 3.8$%. The expression of p53 in this group of tumours did not differ significantly from cultures of normal urothelium from patients with bladder carcinoma ($P =$ 0.2632) or from paraffin-embedded specimens of normal urothelium from TCC patients ($P = 0.398$).

### Discussion p53 has been identified as having a pivotal role in human carcinogenesis (Harris and Hollstein, 1993). This study was aimed at confirming the overexpression of p53 in TCC observed by others (Sidransky *et al.*, 1991; Fujimoto *et al.*, 1992) and determining if this overexpression of p53 could also be found in normal urothelium from patients with TCC. This finding would support an early involvement of p53 malfunction in premalignant urothelium and a 'field change' model for TCC. The results for staining of 22 invasive tumours with p53-D07 are similar to those of Wright *et al.* (1991), who found that 64% ($n = 33$) of invasive tumours stained moderately or strongly positive with PAb 240 or PAb 1801. The use of an arbitrary scale for staining intensity in Wright's study prevents a direct comparison with our results. p53-D07 was used to stain for p53 in the studies of paraffin-embedded invasive TCC and normal urothelium prior to culture. This antibody detects both wild and mutant conformations of the protein; however, expression in paraffin-embedded tissue is thought to be exclusively due to the mutated conformation of the protein (Lane and Benchimol, 1990). p53 overexpression in TCC has been shown to arise from genetic mutation rather than post-translational effects. The status of p53 protein expression in premalignant urothelium is unclear but, given the current views on the role of p53 control of growth, DNA damage response and apoptosis (reviewed in Cohen, 1993), it would seem logical that alterations in p53 protein function as opposed to underlying gene sequence could be expected at an early stage of carcinogenesis.

The results presented in this paper indicate that p53 overexpression is found in normal urothelium in patients with invasive TCC. Since specimens were taken from sites remote from the carcinoma (usually the ureters from cystectomy patients) and screened histologically for evidence of carcinoma or dysplasia, it is unlikely that the observed expression of p53 was due to the inadvertent inclusion of malignant cells in the explant specimen. The results, therefore, strongly support a field change model for TCC rather than a clonal model since it is hard to conceive of successful implantation of malignant tumour clones throughout every area of the urothelium accounting for 20–50% of cells in those areas, without then having any histologically recognisable or phenotypic changes. Specimens of normal urothelium from patients with invasive TCC were assessed for expression of p53 with both p53-D07, in intact tissue, and p53-240 following cell culture of specimens. While the expression of the protein was substantially higher following cell culture (mean percentage positive cells 46.4% *vs* 28.2%), this difference was not significant when analysed using a paired Student *t*-test ($P = 0.1916$). There was no correlation between p53 expression and either stage or grade of the primary tumour.

This difference in expression between the two methods is not surprising since the two antibodies are directed against different epitopes. The cell cycle-dependent expression of p53 would also be expected to lead to detection of higher levels in growing cultures as opposed to differentiated tissue.

p53-positive cells appeared to have an enhanced growth rate and should consequently be present in higher numbers following tissue culture. None of the 60 specimens from tumour-free patients was found to express p53 when examined prior to culture with p53-D07, yet 23% showed >5% of cells positive when stained with p53-240 following tissue culture. This may be due to a growth advantage since the percentage of cells positive for p53 showed a linear correlation with growth in this subset of normal cultures. A similar relationship between cell growth in tissue culture and p53 expression was not observed in cell cultures from tumour-bearing patients, however the rate of cell growth was significantly higher in this group and conditions *in vitro* or the natural proliferation capacity of the cells may have limited cell growth.

The presence of p53 protein in tumour-free cell cultures, but not in the corresponding paraffin-embedded specimens, might be accounted for by clonal expansion of a small number of p53-positive cells in the original explant. This is, however, unlikely because there was no evidence in the cultures or sections of clonal expression of p53. It is also difficult to see how a 2-fold increase in cell number could account for a 10+-fold increase in cells that overexpress p53 in the 'high' *vs* the 'low' p53-expressing group. If clonal expansion is not the cause of the normal high p53 expression group, then this group is interesting but without explanation. Our current hypothesis is that the members of the high normal group are expressing high levels of (probably) wild-type p53 owing to fast proliferation or accumulated DNA damage from environmental carcinogen exposure.

Despite this subgroup of tumour-free patients who expressed p53 following cell culture, there was a significant difference in the mean percentage positive cells between tumour-free and TCC normal urothelial cell cultures. This overexpression of p53 protein has not been previously described in normal urothelium from patients with invasive TCC and implicates p53 dysfunction in the early stages of carcinogenesis in this disease. The association with increased cell growth suggests a consequent disturbance in control of cellular proliferation. A similar phenomenon was observed by Farsund *et al.* (1984) when studying cell cycle distribution in urothelium at sites distant from TCC, it was found that a higher proportion of cells were in S or $G_2$ when compared to tumour-free patients. Since wild-type p53 inhibits cellular growth (Sidransky *et al.*, 1992), an abnormality in cell proliferation, associated with aberrant p53 expression, is not surprising. Our studies, however, cannot distinguish whether this association is causal or incidental.

Such conformational variants of the protein have been shown to bind p53-240 (Farsund *et al.*, 1984). The nature of the p53 protein detected by p53-240, following cell culture, is uncertain. While the antibody is specific for the mutant form in immunoprecipitation studies, immunohistochemical staining may detect stable, wild-type conformational variants (Gannon *et al.*, 1990). Stable, wild-type p53 protein has recently been observed in normal cells of a cancer family member without evidence of genetic mutation (Kern *et al.*, 1992). In addition, marrow blast cells from normal individuals have been shown to express wild-type protein which is identified by the p53-240 antibody (Ramel *et al.*, 1992). The abnormal expression of p53 seen on tissue culture may, therefore, not necessarily reflect a genetic mutation.

Abnormal p53 expression is being increasingly recognised in dysplastic, premalignant conditions. In Barrett's oesophagus, increasing frequency of gene product expression has been observed with increasing dysplasia (Barnes *et al.*, 1992; Rivas *et al.*, 1992; Kaklamanis *et al.*, 1993). We have previously shown that, following cell culture, increased p53 protein is expressed in cells from adjacent normal mucosa in oesophageal carcinoma (Mothersill *et al.*, 1994). A similar association between abnormal p53 expression and dysplasia has been made in colonic adenomas; however, the protein was observed in focal areas of dysplasia rather than throughout the adenoma (Kawasaki *et al.*, 1992). The absence of p53 in non-dysplastic areas of colorectal adenomas supports the hypothesis that abnormalities in p53 occur in the transition from adenoma to carcinoma and is thus a relatively late step in carcinogenesis (Kawasaki *et al.*, 1992). Our study shows that abnormalities in p53 take place at an earlier stage in urothelial carcinogenesis.

Histologically normal urothelial cells, at sites distant from the tumour, were found to express the protein, implicating p53 as part of a more generalised field change in patients with invasive TCC. Expression of the protein was also associated with increased cellular proliferation *in vitro*. These findings suggest that aberrant p53 expression in normal urothelium may be predictive of future carcinogenesis.

### References

BARNES DM, HANBY AM, GILLETT CE, MOHAMMED S, HODGSON S, BORROW LG, LEIGH IM, PURKIS T, MACGEOCH C, SPURR NK, BARTEK J, VOJTESEK B, PICKSLEY SM & LANE DP. (1992). Abnormal expression of wild type p53 protein in normal cells of a cancer family patient. *Lancet*, **340**, 259-263.

COHEN JJ. (1993). Apoptosis. *Immunol. Today*, **14**, 126-130.

ESRIG D, SPRUCK CH, NICHOLS PW, CHALWUN B, STEVEN K, GROSHEN S, CHEN SC, SKINNER DG, JONES PA AND COTE RJ. (1993). p53 Nuclear protein accumulation correlates with mutations in the p53 gene, tumour grade and stage in bladder cancer. *Am. J. Pathol.*, **143**, 1289-1397.

FARSUND T, HOESTMARK JG AND LAERUM OD. (1984). Relationship between flow cytometric DNA distribution and pathology in human bladder cancer. *Cancer*, **54**, 1771-1777.

FUJIMOTO K, YAMADA Y, OKAJIMA E, KAKIZOE T, SASAKI H, SUGIMURA T AND TERADA M. (1992). Frequent association of p53 gene mutation in invasive bladder cancer. *Cancer Res.*, **52**, 1393-1398.

FURIHATA M, INOUE K, OHTSUKI Y, HASHIMOTO H, TERAO N AND FUJITA Y. (1993). High risk human papilloma virus infections and overexpression of p53 protein as prognostic indicators in transitional cell carcinoma of the urinary bladder. *Cancer Res.*, **53**, 4823-4827.

GANNON JV, GREAVES R, IGGO R AND LANE DP. (1990). Activating mutations in p53 produce a common conformational effect. A monoclonal antibody specific for the mutant form. *EMBO J.*, **9**, 1595-1601.

HABUCHI T, OGAWA O, KAKEHI Y, OGURA K, KOSHIBA M, SUGIYAMA T AND YOSHIDA O. (1992). Allelic loss of chromosome 17P in urothelial cancer: strong association with invasive phenotype. *J. Urol.*, **148**, 1595.

HARRIS C AND HOLLSTEIN M. (1993). Clinical implications of the p53 tumour suppressor gene. *N. Engl. J. Med.*, **392**, 1318.

HOLLSTEIN M, SIDRANSKY D, VOGELSTEIN B AND HARRIS CC. (1991). p53 mutations and human cancer. *Science*, **253**, 49-53.

HSU SM, RAINE L AND FANGER H. (1981). Use of avidin-biotin-peroxidase complex (ABC) in immunoperoxidase techniques. *J. Histochem Cytochem.*, **29**, 577.

JONES RF, MATUSZYK J, DEBIEC-RYCHTEN M AND WANG CY. (1994). Mutation and altered expression of p53 genes in experimental rat bladder tumour cells. *Mol. Carcinogen.*, **9**, 95-104.

KAKLAMANIS L, GATTER KC, MORTENSEN N, BAIGRIE RJ, HERYET A, LANE DP AND HARRIS AL. (1993). p53 expression in colorectal adenomas. *Am. J. Pathol.*, **142**, 87-93.

KASTEN MB, ONYEWERE O, SIDRANSKY D, VOGELSTEIN B AND CRAIG RW. (1991). Participation of p53 protein in the cellular response to DNA damage. *Cancer Res.*, **51**, 6304-6311.

KAWASAKI Y, MONDEN T, MORIMOTO H, MUROTANI M, MIYOSHI Y, KOBAYASHI T, SHIMANO T AND MORI T. (1992). Immunohistochemical study of p53 expression in microwave fixed, paraffin-embedded sections of colorectal carcinoma and adenoma. *Am. J. Clin. Pathol.*, **97**, 244-249.

KERN SE, PIETENPOL JA, THIAGALINGAM S, SEYMOUR A, KINZLER KW AND VOGELSTEIN B. (1992). Oncogenic forms of p53 inhibit p53-regulated gene expression. *Science*, **256**, 824-829.

LAMBKIN H, MOTHERSILL C, CHIN D, DUFFY M, SHEEHANK AND PARFREY NP. (1994). p53 Immunohistochemistry in breast adenocarcinoma - a panel of antibodies compared with SSCP. *J. Pathol.* (in press).

LANE DP. (1992). p53, guardian of the genome. *Nature*, **358**, 15-16.

LANE DP AND BENCHIMOL S. (1990). p53: oncogene or anti-oncogene? *Genes and Dev.*, **4**, 1-8.

LIPPONEN PK. (1993). Over-expression of p53 nuclear oncoprotein in transitional-cell bladder carcinoma and its prognostic value. *Int. J. Cancer*, **53**, 365-370.

MOTHERSILL C, CUSACK A, McDONNELL M, HENNESSY TP AND SEYMOUR CB. (1988). Differential response of normal and tumour oesophageal explant cultures to radiation. *Acta Oncol.*, **27**, 275-280.

MOTHERSILL C, SEYMOUR CB, HARNEY J AND HENNESSY TP. (1994). Expression of high levels of stable p53 and of cmyc in cultured human epithelial tissue following carcinogen challenge using $^{60}$Co irradiation. *Radiat. Res.*, **137**, 317-322.

PETERSEN I, OHGAKI H, LUDEKE BI AND KLEIHUES P. (1993). p53 Mutation in phenacetin-induced urothelial carcinomas. *Vehr. Dtsch. Ges. Pathol.*, **77**, 252-255.

RAMEL S, REID BJ, SANCHEZ CA, BLOUNT PL, LEVINE DS, NESHAT K, HAGGITT RC, DEAN PJ, THOR K AND RABINOVITCH PS. (1992). Evaluation of p53 expression in Barrett's esophagus by two-parameter flow cytometry. *Gastroenterology*, **102**, 1220-1228.

RIVAS CI, WISNIEWSKI D, STRIFE A, PEREZ A, LAMBEK C, BRUNO S, DARZYNKIEWICZ Z AND CLARKSON B. (1992). Constitutive expression of p53 protein in enriched normal human marrow blast cell populations. *Blood*, **79**, 1982-1986.

SARKIS AS, DALBAGNI G, CORDON-CARDO C, ZHANG ZF, SHEINFELD J, FAIR WR, HERR HW AND REUTER. (1993). Nuclear over-expression of p53 protein in transitional cell bladder carcinoma: a marker for disease progression. *J. Natl Cancer Inst.*, **85**, 53-59.

SIDRANSKY D AND MESSING E. (1992). Molecular Genetics and biochemical mechanisms in bladder cancer. Oncogenes, tumour suppressor genes and growth factors. *Urol. Clin. N. Am.*, **19**, 629-639.

SIDRANSKY D, VON ESCHENBACH A, TSAI TC, JONES P, SUMMERHAYES I, MARSHALL F, PAUL M, GREEN P, HAMILTON SR, FROST P AND VOGELSTEIN B. (1991). Identification of p53 gene mutation in invasive bladder cancers and urine samples. *Science*, **252**, 706-709.

SPRUCK CH, RIDEOUT WM, OLUMI AF, OHNESEIT PF, YANG AS, TSAI YC, NICHOLS PW, HORN T, HERMANN GG AND STEVEN K. (1993). Distinct pattern of p53 mutations in bladder cancer: relationship to tobacco usage. *Cancer Res.*, **53**, 1162-1166.

WERNESS BA, LEVINE AJ AND HOWLEY PM. (1990). Association of human papilloma virus types 16 and 18 E6 proteins with p53. *Science*, **248**, 76-79.

WRIGHT C, MELLON K, JOHNSTON P, LANE DP, HARRIS AL, HORNE CH, AND NEAL DE. (1991). Expression of mutant p53, c-erbB-2 and epidermal growth factor receptor in transitional cell carcinoma of the human urinary bladder. *Br. J. Cancer*, **63**, 967-970.